# Oral Health in Tanzania: Unmasking Its Neglected Dimension

**DOI:** 10.1002/puh2.70000

**Published:** 2024-09-13

**Authors:** Majani Edward, Daniel Agyapong, Innocent Kitandu Paul, Ibrahim Idris, Gbassara Koulagna Boris, Nathan Ezie Kengo, Emelia Azeyele Kpiebaya, Shuaibu Saidu Musa, Don Eliseo Lucero‐Prisno

**Affiliations:** ^1^ Department of Public Health St. Francis University College of Health and Allied Sciences Morogoro Tanzania; ^2^ Faculty of Bioscience University for Development Studies Nyankpala Ghana; ^3^ Faculty of Medicine Catholic University of Health and Allied Sciences Mwanza Tanzania; ^4^ College of Medicine Kaduna State University Kaduna Nigeria; ^5^ Faculty of Medicine and Biomedical Sciences University of Yaounde I Yaounde Cameroon; ^6^ Faculty of Medicine and Biomedical Sciences University of Garoua Garoua Cameroon; ^7^ Research Division Winners Foundation Yaounde Cameroon; ^8^ Faculty of Medicine Department of Pharmacology and Therapeutics American University of Beirut Beirut Lebanon; ^9^ Faculty of Medicine School of Global Health Chulalongkorn University Bangkok Thailand; ^10^ Department of Nursing Science Ahmadu Bello University Zaria Nigeria; ^11^ Faculty of Public Health and Policy Department of Global Health and Development London School of Hygiene and Tropical Medicine London UK; ^12^ Office for Research, Innovation and Extension Services Southern Leyte State University Sogod Southern Leyte Philippines; ^13^ Center for University Research University of Makati Makati Philippines

**Keywords:** neglected dimension, oral health, Tanzania, unmask

## Abstract

The World Health Organization's definition of oral health underscores its holistic nature encompassing physiological, psychosocial, and functional dimensions. The current estimates of oral diseases in Tanzania and around the world underscore the urgency of intervention, particularly in light of rising sugar consumption trends. The unique challenges facing Tanzania, including inadequate knowledge, limited infrastructure, and disparities in oral healthcare access, are analyzed within the broader context of sub‐Saharan Africa's health priorities. This article addresses the multifaceted challenges of oral health neglect in Tanzania, emphasizing the imperative need for a comprehensive and integrated approach. The discussion offers a detailed exploration of determinants contributing to oral health neglect, spanning socioeconomic, behavioral, and commercial factors, with a focus on their implications for Tanzanian communities. Recommendations are presented as a strategic roadmap, encompassing public health education, integrated healthcare services, government intervention, educational programs, community engagement, financial accessibility, and research initiatives. By synthesizing these recommendations into a cohesive framework, a proactive and collaborative approach to mitigate the consequences of oral health neglect in Tanzania, emphasizing the need for transformative policies and cultural sensitivity, is therefore envisioned.

## 1 Introduction

Oral health, as defined by the World Health Organization (WHO), encompasses the condition of the mouth, teeth, and orofacial structures that allow individuals to perform basic bodily functions like eating, breathing, and speaking. It also includes psychosocial elements, such as self‐esteem, well‐being, and the ability to interact with others and complete daily tasks without experiencing pain, discomfort, or shame [1]. It is estimated that around 3.5 billion people worldwide suffer from oral diseases, which caused roughly 180,000 deaths in 2020 [2]. Tanzania, a country in East Africa, has a population of roughly 45 million [3]. It experiences low‐to‐moderate levels of oral disease prevalence [4]. A 2022 WHO report highlighted an alarming oral health situation in Tanzania in 2020–2022, indicating a critical need for intervention [5]. The emergence of this situation could be attributed to inadequate knowledge on oral health and neglect among Tanzanians. This is evidenced by the lack of adequate oral healthcare infrastructure and limited oral health personnel, as Tanzania's dentist‐to‐patient ratio is 1:360,000, which is lower than the WHO standard [1]. To prevent oral illnesses, and maintain good oral hygiene and overall health, it is essential to address the neglect of oral healthcare in Tanzania.

## 2 Oral Diseases in Tanzania

In 2019, the WHO estimated that nearly half of the world's population suffers from various forms of oral disease [6]. In March 2023, WHO Africa reported a noticeable increase in oral diseases of more than 250 million cases over the last 3 decades in the region [7], which is aggravated by various challenges aside from poor health systems. The workforce in the oral medicine field is disproportionately low. Tanzania has a high prevalence of untreated dental caries of permanent teeth among children older than 5 years. This is more than 30% compared to the other age groups [8]. The main risk factor for this high prevalence is an overall change in lifestyle among the children, such as consumption of manufactured sweets and lack of awareness on oral health. These factors contribute significantly to the high incidence of dental caries. The most affected populations are those who reside in the rural parts of the country [9].

## 3 Determinants of Oral Health Neglect

Neglect in oral health is a multifaceted issue influenced by many factors. The complexity of this problem is further underscored by varying perspectives on its definition, response, and perceived nature—whether categorized as active or passive. Determinants affecting oral health in Tanzania can be classified into three main groups: socioeconomic, behavioral, and commercial factors associated with risk. Socioeconomic disparities are evident in Tanzania, with notable variations based on professions of parents and income levels. For example, children from more affluent families are more likely to receive orthodontic treatment, highlighting the impact of societal inequalities. Additionally, parental influence, particularly from educated mothers, plays a decisive role, serving as a positive factor in oral hygiene practices. Conversely, limited parental knowledge about oral health increases the likelihood of poor dental care in children.

A noteworthy variable is geographical access. Urban areas in Tanzania offer better access to dental services than rural areas, creating regional disparities in oral health. Challenges such as the absence of basic oral care resources in certain areas can also result from unfavorable geographical conditions. Daily habits have a major impact on oral health, with the frequency of tooth brushing, parental supervision, and the use of fluoride toothpaste being fundamental elements in preventing cavities. Parental influence, especially that of mothers, serves as a crucial factor in shaping children's oral hygiene habits and dietary choices. Commercial influences and modifiable lifestyle choices are noteworthy contributors to oral health issues. The marketing of sugar‐rich products **i**ndirectly impacts oral health, thus increasing the risk of cavities and other dental complications. Lifestyle habits such as smoking and alcohol consumption are closely associated with various oral diseases, whereas dietary choices, particularly a diet rich in added sugars, act as triggers for cavities in both children and adults. Cultural and religious beliefs play a significant role in shaping attitudes and practices regarding oral health. For example, dental esthetics are associated with an attractive appearance, motivating some community members to seek professional dental care. Access to dental care may be influenced by affiliation to a religious community [10].

## 4 Barriers to Oral Care

A major challenge with the healthcare system of countries with challenged economies like Tanzania is the pervasive out‐of‐pocket payment for health services. Reasons advanced by those not seeking emergency dental treatment despite the need include insufficient funds to cover treatment costs (27.9%), self‐medication (17.6%), the belief that pain would disappear eventually (15.7%), and insufficient funds to cover transportation to the dental clinic (15.0%) [11]. In a study conducted among 1106 individuals, over 90% of the participants were aware that the health center or dispensary offered emergency dental care [12]. A positive history of dental disorders in the household was found to be the main predictor of the utilization of emergency oral healthcare [12]. Such attitudes where patients are highly reluctant to decide on seeking consult, or having difficulties accessing facilities, and the fear or delay in receiving treatment, pose an obstacle to the advancement of oral health initiatives. This poses a problem in the evaluation of effectiveness and needs assessment so as to meet the actual challenge in improving oral health.

Within the realm of restorative care, the major barriers in Tanzania are the lack of guidance from the dentist, a lack of understanding about restorative dentistry, distorted views and misconceptions, the absence of regular dental check‐up practices, the potential for discomfort when seeking restorative therapy, and prior dental care experience [13]. Hence, identifying and addressing barriers to improving oral health initiatives are paramount. These need to cut across economic, cultural, and professional reshaping. A perceived impediment to the use of oral healthcare was an inability to pay for the services, even when accessible, thus raising the need to address these financial constraints.

## 5 Impact of Oral Health Neglect

Oral health is important to overall health and well‐being; unfortunately, it is often neglected in low‐income countries like Tanzania. Neglected oral diseases, such as dental caries, periodontal disease, noma, and oral cancer, can have serious consequences to individuals and communities. These conditions directly affect their quality of life, productivity, and social interactions [14]. People most affected and burdened often have the least access to services, resulting in unmet treatment needs and poor oral hygiene practices [15]. There is also a lack of integration of oral health services in the primary healthcare system and low awareness and knowledge among the public about the importance and prevention of oral diseases [16]. Moreover, oral diseases have been linked to an increased risk of conditions like diabetes, cardiovascular disease, and respiratory infections. It is important to note that the relationship between oral health problems and these systemic diseases is bidirectional; thus, they interact and influence each other. The economic burden becomes substantial, as untreated oral health issues lead to decreased productivity due to absenteeism and impaired performance at work or in school [17].

## 6 Conclusion

Addressing the complex issue of oral health neglect in Tanzania necessitates a multifaceted and integrated approach. Comprehensive public health education campaigns are crucial in raising awareness about the significance of oral health and its impact on overall well‐being. There is a pressing need to integrate oral health into the primary healthcare system, thus fostering cross‐disciplinary collaboration among healthcare professionals to ensure a holistic approach to healthcare. Government intervention is imperative to allocate resources and funding to enhance oral healthcare infrastructure, thus mitigating the shortage of oral health personnel, and ensuring equitable accessibility to dental services, particularly in rural areas. Educational programs and community engagement programs tailored to accommodate cultural and religious beliefs are pivotal in empowering individuals to take proactive measures for oral health. Financial accessibility must be addressed through initiatives such as subsidizing treatment costs and establishing community‐based oral health insurance schemes. The need to conduct research and regularly collect epidemiological data will be instrumental in understanding specific oral health needs, tracking trends, and shaping evidence‐based interventions. By implementing these comprehensive recommendations, Tanzania can proactively work toward reducing the burden of oral diseases, improving overall health outcomes, and fostering a healthcare landscape where oral health is prioritized and well integrated.

## Author Contributions

Majani Edward conceptualized the paper, wrote the original draft, reviewed, and edited while other authors wrote the original draft except Don Eliseo who participated in the review and editing.

## Ethics Statement

The authors have nothing to report.

## Consent

The authors have nothing to report.

## Conflicts of Interest

Shuaibu Saidu Musa is a Youth Editorial Board member of Public Health Challenges and a coauthor of this article. Don Eliseo Lucero‐Prisno III is the Chief Editor of Public Health Challenges and a coauthor of this article. They are therefore excluded from all editorial decision‐making related to the acceptance of this article for publication.

## Data Availability

Data were collected from the prior findings.
